# Postoperatives Schmerzerleben nach proximaler Femurfraktur bei Demenz

**DOI:** 10.1007/s00482-021-00619-5

**Published:** 2022-01-17

**Authors:** Jens Felix Wagner, Henning Cuhls, Martin Mücke, Rupert Conrad, Lukas Radbruch, Roman Rolke

**Affiliations:** 1 Akutgeriatrie und Tagesklinik, Helios Klinikum Bonn/Rhein-Sieg, Bonn/Rhein-Sieg, Deutschland; 2 Klinik und Poliklinik für Palliativmedizin, Universitätsklinikum Bonn, Bonn, Deutschland; 3 Institut für Digitale Allgemeinmedizin, Medizinische Fakultät, RWTH Aachen University, Aachen, Deutschland; 4 Zentrum für Seltene Erkrankungen Aachen (ZSEA), Medizinische Fakultät, RWTH Aachen University, Aachen, Deutschland; 5 Klinik und Poliklinik für Psychosomatische Medizin und Psychotherapie, Universitätsklinikum Bonn, Bonn, Deutschland; 6 Zentrum für Palliativmedizin, Helios Klinikum Bonn/Rhein-Sieg, Bonn/Rhein-Sieg, Deutschland; 7 Klinik für Palliativmedizin, Medizinische Fakultät, RWTH Aachen University, Aachen, Deutschland

**Keywords:** Schmerzempfindungsskala, Quantitative sensorische Testung, Somatosensorisches Nervensystem, Sensibilitätsprüfung, Geriatrie, Pain sensation scale, Quantitative sensory testing, Somatosensory nervous system, Sensory examination, Geriatrics

## Abstract

**Hintergrund:**

Die vorliegende Studie verfolgte das Ziel, das postoperative Schmerzerleben bei kognitivem Defizit unter besonderer Berücksichtigung der sensorischen oder affektiven Schmerzqualität zu erfassen.

**Methodik:**

19 Patienten mit normaler Kognition bis hin zu kognitiven Auffälligkeiten im Screening-Fragebogen DemTect wurden bezüglich ihres postoperativen Schmerzerlebens nach proximaler Femurfraktur untersucht. Als Untersuchungsinstrumente kamen die numerische Ratingskala (NRS), der Kognitionsfragebogen DemTect, der Schmerzempfindungsfragebogen (SES) sowie eine quantitative sensorische Testung (QST) zum Einsatz.

**Ergebnisse:**

Das Alter der Patienten lag im Mittel ± SD bei 83,8 ± 10,0 Jahren. Von 19 Patienten lagen bei 6 (31,6 %) normale kognitive Fähigkeiten vor, bei 4 Patienten (21,1 %) ergaben sich Hinweise auf leichte kognitive Einschränkungen, bei 9 Patienten (47,4 %) ergab sich der Verdacht auf Vorliegen einer Demenz. Die mittlere postoperative Schmerzintensität (NRS) betrug 4,0 (1,6). Die berichteten Schmerzintensitäten unterschieden sich bei vergleichbarer analgetischer Therapie nicht zwischen den drei Patientengruppen mit unterschiedlicher kognitiver Beeinträchtigung und den ersten drei postoperativen Behandlungstagen. Es zeigten sich zwischen den Gruppen keine statistisch signifikanten Unterschiede für die sensorischen oder affektiven Summenscores der Schmerzempfindungsskala.

Die QST-Parameter Tiefenschmerz (PPT), oberflächlicher mechanischer Schmerz nach Nadelreiz (MPT) sowie die oberflächliche Sensibilität für leichte Berührungsreize (MDT) zeigten eine signifikant gesteigerte Empfindlichkeit der operierten Seite. Für das Vibrationsempfinden (VDT) konnten keine Unterschiede zwischen operierter und gesunder Extremität nachgewiesen werden.

**Diskussion:**

Das postoperative Schmerzerleben unterscheidet sich nicht zwischen Patienten mit normaler und deutlich eingeschränkter Kognition. Die quantitative sensorische Testung zeigte mechanische Hyperalgesien im operierten Areal. Die Studie weist auf die Wichtigkeit einer adäquaten postoperativen Schmerzversorgung auch bei Betroffenen mit Demenz hin.

## Abstract (en)

**Background:**

The present study aimed to assess the postoperative pain experience in cognitive deficit patients with special reference to sensory or affective pain quality.

**Methods:**

Nineteen patients with normal cognition up to cognitive impairments according to the DemTect screening-tool were studied regarding their postoperative pain experience after proximal femur fracture. The numerical rating scale (NRS), the cognitive DemTect questionnaire, the pain sensation questionnaire (SES), and a quantitative sensory test (QST) were used as examination instruments.

**Results:**

The mean ± SD age of the patients was 83.8 ± 10.0 years. Of the 19 patients, 6 (31.6%) had normal cognitive abilities. In 4 patients (21.1%) there were indications of mild cognitive impairments, and in 9 patients (47.4%) the suspicions of the presence of dementia arose. The mean postoperative pain intensity (NRS) was 4.0 (1.6). With comparable analgesic therapy, the reported pain intensities did not differ between the three patient groups with different cognitive impairments and the first three postoperative treatment days. There were no statistically significant differences between the groups for the sensory or affective total scores of the pain sensation scale.

The QST parameters deep pain (PPT), superficial mechanical pain after needle stimulation (MPT), and the superficial sensitivity to light touch stimuli (MDT) showed a significantly increased sensitivity of the operated side. For the sensation of vibration (VDT) no differences between operated and healthy extremities could be proven.

**Discussion:**

The postoperative pain experience does not differ between patients with normal and limited cognition. The quantitative sensory testing showed mechanical hyperalgesia in the operated area. The study points to the importance of adequate postoperative pain management even in those with dementia.

## Hintergrund und Fragestellung

„Altern“, „Demenz“ und „Schmerz“ sind Begriffe, die in unserer Gesellschaft zunehmend an Bedeutung gewinnen. Bei einer prognostizierten Abnahme der Gesamtbevölkerung von 82 Mio. in 2008 auf 73,8 Mio. in 2040 steigt der Anteil älterer Menschen über 65 von 20,4 Mio. auf 32,1 Mio. an (Statistisches Bundesamt, 2009). Mit der höheren Lebenserwartung nimmt die Prävalenz von Krankheiten und funktionellen Einschränkungen zu. So führen beispielsweise Osteoporose und vermehrte Sturzneigung bedingt durch funktionellen Abbau, Sehschwäche, Muskelschwäche, Gang- und Gleichgewichtsstörungen sowie zentral wirksame Medikamente zu erhöhter Frakturneigung. Das Risiko, eine Femurfraktur zu erleiden, ist bei dementen Patienten bis zu dreimal höher als bei älteren Patienten mit normalem kognitivem Status [11, 41].

Die angemessene Beurteilung von Schmerzen bei Menschen mit mittelgradiger Demenz stellt für die betreuenden Pflegefachkräfte und Ärzte eine Herausforderung dar [12, 22]. So werden stärkere Schmerzen eher unterschätzt, leichtere Schmerzen eher überschätzt [8, 40]. Patienten mit Alzheimer-Demenz erhielten in Pflegeeinrichtungen nur ein Drittel der Morphindosis im Vergleich mit Patienten, die einen normalen kognitiven Status aufwiesen [7, 10]. Nach operativer Versorgung einer Femurfraktur zeigten demente Patienten mehr Schmerzen als Vergleichspersonen [30]. Als Folge eines unzureichenden Schmerzmanagements bei Demenz können sich Schlafstörungen, Depressionen und Ängste entwickeln [25]. Auch die Entwicklung eines Delirs kann durch einen intensiven postoperativen Schmerzzustand begünstigt werden [27]. Eine adäquate Schmerztherapie kann zu weniger postoperativen Komplikationen, einer kürzeren Verweildauer im Krankenhaus, einer besseren Lebensqualität sowie reduzierter Morbidität und Mortalität führen [29, 36].

Bislang fehlen Untersuchungen zum postoperativen Schmerzerleben von dementen im Vergleich mit kognitiv unbeeinträchtigten Patienten.

Hieraus leiten sich die konkreten Fragestellungen der vorliegenden Studie ab:

1. Zeigen Betroffene mit Demenz im Vergleich mit kognitiv Unbeeinträchtigten eine veränderte postoperative Stärke der erlebten Ruheschmerzen?
2. Haben Menschen mit Demenz ein verändertes sensorisches oder affektives Schmerzerleben?
3. Wie unterscheidet sich das sensorische Profil (hier erhoben mit mechanischen Testreizen) bei Patienten ohne und mit kognitivem Defizit im Vergleich der operierten und kontralateralen Seite nach Hüftendoprothese?

## Methodik

In der vorliegenden Studie wurde das postoperative Schmerzerleben nach Femurfraktur untersucht. Anhand des Demenz-Screening-Fragebogens „DemTect“ [20] wurden die Patienten in drei Kognitionsgruppen kategorisiert: normale Kognition, leichtes kognitives Defizit und Verdacht auf Demenz. Mittels quantitativer sensorischer Testung (QST) wurde die Schmerzempfindlichkeit der operierten Hüfte mit dem nicht operierten, kontralateralen Bereich verglichen. Ein modifizierter SES-Schmerzfragebogen (SES = Schmerzempfindungsskala) [15] wurde verwendet, um die Patienten zum aktuellen postoperativen Schmerzerleben mithilfe sensorischer und affektiver Deskriptoren zu befragen.

### Patientenkollektiv

In die Studie wurden 19 Patienten mit einer proximalen Femurfraktur nach ausführlicher schriftlicher und mündlicher Aufklärung eingeschlossen, die eine operative Versorgung in Form eines endoprothetischen Ersatzverfahrens oder eines Osteosyntheseverfahrens erhielten. Die Studie mit der Ethik-Antragsnummer 261/12 an der Medizinischen Fakultät der Universität Bonn erfolgte nach den Grundsätzen guter klinischer Praxis (GCP) unter Einhaltung der Deklaration von Helsinki [2].

Einschlusskriterien waren ein Alter über 60 Jahre, eine frisch aufgetretene proximale Femurfraktur, Ruheschmerzen ≥ 2 von 10 auf der numerischen Ratingskala (NRS) sowie die schriftliche Einwilligung zur Teilnahme an der Studie durch den Patienten oder im Fall reduzierter Kognition durch einen Bevollmächtigten. Ausschlusskriterien waren Migräne oder Rückenschmerzen in den letzten acht Wochen oder Erkrankungen, die zu einer Veränderung der Sensibilität gegenüber den Testreizen führen, sowie Hautveränderungen, die eine Anwendung der QST-Geräte einschränken könnten. Außerdem wurden Patienten mit einem postoperativen Delir von der Studienteilnahme ausgeschlossen.

### Studienablauf

Durchgeführt wurde die Untersuchung in den Abteilungen für Chirurgie und Geriatrie des Helios Klinikums (vormals Malteser Krankenhaus) Bonn/Rhein-Sieg vom 11.03.2013 bis zum 03.06.2013. Die Untersuchungen erfolgten im Rahmen einer klinischen monozentrischen Beobachtungsstudie. Alle Patienten erhielten nach dem operativen Eingriff eine auf die individuelle Schmerzangabe abgestimmte und nicht standardisierte analgetische Therapie. Keiner der Patienten erhielt ein Regionalanästhesieverfahren. Alle Testungen (NRS, DemTect, SES, QST) erfolgten durch denselben Untersucher (JFW) innerhalb der ersten drei postoperativen Tage zwischen 10:00 und 16:00 Uhr.

### Anamnese, klinische Untersuchung und Erfassung der aktuellen Schmerzstärke

Nach Anamneseerhebung fanden eine symptombezogene klinische Untersuchung (Wundinspektion) sowie die Beurteilung des klinischen Gesamtzustands statt. Im Anschluss erfolgte die Abfrage der zum Untersuchungszeitpunkt empfundenen Ruheschmerzen mithilfe einer numerischen Ratingskala (NRS 0–10; 0 = „kein Schmerz“; 10 = „maximal vorstellbarer Schmerz“).

### Erfassung des kognitiven Status mittels DemTect-Fragebogen

Der DemTect-Fragebogen erfasst verschiedene kognitive Dimensionen: verbales Gedächtnis, Wortflüssigkeit, intellektuelle Flexibilität und Aufmerksamkeit [20]. Die Rohwerte des Tests wurden in altersbezogene Testwerte umcodiert. Dabei dient der DemTect als Screening und nicht als diagnostisches Instrument mit dem Ergebnis eines Summenscores: 0–8 = Verdacht auf Demenz; 9–12 = leichte kognitive Beeinträchtigung; 13–18 = normale Kognition.

### Schmerzempfindungsskala (SES)

Der hier verwendete modifizierte SES-Fragebogen erfasst die Ausprägung von 24 Schmerzdeskriptoren zur Charakterisierung des aktuellen Schmerzerlebens [15]. Der Patient bewertet jeden Deskriptor im affektiven und sensorischen Teil der Schmerzempfindungsskala auf einer 4‑Punkte-Skala. Als Anpassung zum Originalfragebogen wurde in dieser Untersuchung das Fehlen eines Schmerzmerkmals mit dem Wert „0“ („trifft nicht zu“) bewertet. Eine maximale Ausprägung wurde mit der Zahl „3“ („trifft genau zu“) bewertet. Die Werte für die Globaldimension SES-affektiv und SES-sensorisch wurden anschließend durch Summation der Testwerte der einzelnen Deskriptoren ermittelt.

### Quantitative sensorische Testung (QST)

Die quantitative sensorische Testung ist eine formalisierte und standardisierte klinische Sensibilitätsprüfung mit kalibrierten Testreizen [31]. Die Untersuchung wurde anhand des DFNS-Protokolls (Deutscher Forschungsverbund Neuropathischer Schmerz) durchgeführt [38, 39]. Da den für die Operation anstehenden Patienten für die QST-Untersuchung kein Transport in ein Untersuchungslabor zumutbar war, wurden nur mechanische Tests im Patientenzimmer durchgeführt und auf eine schwer transportable PC-gestützte Thermotestung verzichtet. Die Testung wurde am ventralen Oberschenkel am Übergang vom proximalen zum mittleren Drittel der Strecke zwischen der Spina iliaca anterior superior und der Patella durchgeführt. Die Testung erfolgte in einem ausreichenden Abstand von ca. 5–8 cm zur Operationswunde. Zunächst erfolgte als Referenz die QST-Untersuchung der gesunden Seite (Kontrollareal), anschließend der operierten Seite (Testareal). Dabei kamen vier Verfahren mit mechanischen Testreizen zum Einsatz: die mechanische Detektionsschwelle (MDT = mechanical detection threshold) wurde mittels eines standardisierten Sets modifizierter von „von Frey“-Filamente bestimmt (Optihair_2_-Set, Marstock, Deutschland; 0,25 bis 512 mN) [13]. Die mechanische Detektionsschwelle wurde als geometrischer Mittelwert aus fünf gerade überschwelligen und fünf gerade unterschwelligen Messwerten bestimmt.

Die Untersuchung der mechanischen Schmerzschwelle (MPT = mechanical pain threshold) erfolgte mit Nadelreizstimulatoren (Pinpricks) [4]. Ziel war die Erfassung des ersten schmerzhaften Nadelreizes, der als „spitz“ oder „pieksend“ empfunden wurde. Dabei wurden Nadelreizstimulatoren mit flacher Spitze (0,25 mm Durchmesser; 8 bis 512 mN) verwendet. Als Schmerzschwelle wurde der geometrische Mittelwert von jeweils fünf gerade über- und unterschwelligen Reizstärken berechnet.

Die Vibrationsdetektionsschwelle (VDT = vibration detection threshold) wurde mittels einer standardisierten 64-Hz-Stimmgabel [5, 17] untersucht, die ebenfalls im Abstand von 5–8 cm zur Op.-Wunde mit sehr leichtem Druck auf die Haut aufgebracht wurde. Im Unterschied zum DFNS-QST-Protokoll wurde VDT entsprechend nicht über einer knöchernen Prominenz untersucht. VDT wurde als gerade nicht mehr wahrgenommene Vibrationsempfindung in x/8 als arithmetischer Mittelwert von drei Serien absteigender Stimulusintensitäten angegeben.

Die Druckschmerzschwelle (PPT = pressure pain threshold) wurde mittels eines Druckalgometers (Algometer, Somedic, Schweden) bestimmt. Mit einer gummierten Kontaktfläche von 1 cm^2^ wurden drei Serien zunehmender Reizintensitäten (0,5 kg/cm^2^*s, entspricht ca. 50 kPa/s) bis maximal 20 kg/cm^2^ (ca. maximal 2000 kPa) über der zu untersuchenden Körperregion aufgebracht. Als Druckschmerzschwelle wurde die aus drei Messungen gemittelte Reizintensität der jeweils ersten schmerzhaft drückenden Wahrnehmung bestimmt [23, 37].

### Statistische Auswertungen

Die Auswertung der Schmerzschätzungen sowie der QST-Befunde erfolgte mithilfe des Programms STATISTICA 7.1 (Statsoft Europe, Hamburg, Deutschland). Aufgrund der nicht-parametrischen Verteilung einzelner QST-Parameter wurde nach sekundärer Normalisierung der Daten durch eine Log-Transformation eine Varianzanalyse (ANOVA) für multivariate Analysen durchgeführt. Anschließend folgten Post-Hoc-Tests (LSD-Post-Hoc-Test = least significant difference), um einzelne Kontraste darzustellen. Die QST-Daten wurden grafisch als Rohdaten-Mittelwerte (SEM) dargestellt. Neben dem Vergleich der operierten und Kontrollseite wurde hier die postoperative Schmerzempfindlichkeit auch im Vergleich der Kognitionsgruppen analysiert. Die Zuteilung der Patienten zu den drei Kognitionsgruppen wurde nach Anzahl der erreichten Punkte im DemTect getroffen. Die Daten des SES-Fragebogens wurden deskriptiv und zusätzlich mittels getrennter Faktorenanalysen für die affektiven und sensorischen Schmerzdeskriptoren untersucht. Die Datenextraktion erfolgte mit der Varimax-Methode (einfache Datenrotation). Berücksichtigt wurden nur Faktoren mit einem Eigenwert > 1.

## Ergebnisse

### Patienten

Das Alter der Patienten lag im Mittel ± SD bei 83,8 ± 10,0 Jahren. 84 % der Patienten waren Frauen, 16 % waren Männer. Von den 19 Studienpatienten wurden elf (57,9 %) am ersten, zwei (10,5 %) am zweiten und sechs (31,6 %) am dritten postoperativen Tag untersucht. Bei 16 Patienten lag eine mediale Schenkelhalsfraktur vor (84,2 %), bei zwei Patienten eine subtrochantäre Femurfraktur (10,5 %), bei einem Patienten handelte es sich um eine pertrochantäre Femurfraktur (5,3 %). In zehn Fällen (52,6 %) war die Fraktur auf der rechten Seite lokalisiert, in neun Fällen (47,4 %) auf der linken. In der Tab. 1 sind die Patientendaten nach Kognitionsgruppen mit Alter, Geschlecht, aktuellem NRS und Schmerzmedikation zusammengefasst.

**Table 1:** 

Kognition Gruppe	Kognition Punktezahl DemTect	Alter	Geschlecht	NRS	Opioide (Tagesdosis)	Nichtopioid-Analgetika (Tagesdosis)
1	14	61	m	7	–	Ibuprofen 1800 mg
1	15	62	w	5	–	Ibuprofen 1800 mg
1	18	70	w	3	Tilidin 200 mg	Ibuprofen 1800 mg
1	18	75	w	4	–	Metamizol 2 g
1	14	84	m	3	–	Metamizol 1,5 g
1	16	88	w	7	–	Ibuprofen 1800 mg, Metamizol 2 g
2	10	83	w	2	–	Ibuprofen 1800 mg, Metamizol 4 g
2	9	86	w	2	Tilidin 150 mg	Metamizol 1,5 g
2	10	91	m	4	Tilidin 100 mg	Metamizol 4 g
2	10	92	w	5	Tilidin 100 mg	Metamizol 2 g
3	8	83	w	4	Tilidin 100 mg	Metamizol 2,5 g
3	7	83	w	6	–	Ibuprofen 1200 mg, Metamizol 2 g, Diclofenac 150 mg
3	4	85	w	2	Tilidin 100 mg	Ibuprofen 1800 mg
3	6	89	w	4	–	Metamizol 2 g
3	5	89	w	3	Tilidin 200 mg	Metamizol 4 g
3	8	92	w	2	–	Ibuprofen 1200 mg, Diclofenac 75 mg
3	8	93	w	4	Hydromorphon 24 mg	Flupirtin, 200 mg, Metamizol 2,5 g
3	8	93	w	5	Tilidin 300 mg	Ibuprofen 1800 mg, Metamizol 2 g
3	4	93	w	4	–	Metamizol 2 g

Legende: Kognitionsgruppe nach DemTect: *1* = normale Kognition; *2* = leichtes kognitives Defizit; *3* = Verdacht auf Demenz. *NRS* numerische Ratingskala (0–10; 0 = „kein Schmerz“, 10 = „maximaler vorstellbarer Schmerz“)

### Postoperatives medikamentöses Schmerzmanagement

Alle Patienten erhielten Nichtopioid-Analgetika. Es fanden sich dabei keine signifikanten Dosisunterschiede zwischen den Gruppen. In der Gruppe mit normaler Kognition erhielten die Patienten im Mittel ± SD 1,3 ± 0,5 Schmerzmedikamente, in der Gruppe mit milder kognitiver Beeinträchtigung 2,0 ± 0 und in der Gruppe mit Verdacht auf Demenz 2,0 ± 0,9 Schmerzmedikamente. Als Nichtopioid-Analgetika wurden Ibuprofen (1200 bis 1800 mg/Tag) und Metamizol (1500 bis 4000 mg/Tag) und in je einem Fall Flupirtin (200 mg/Tag) oder Diclofenac (150 mg/Tag) verordnet. 47 % der Patienten wurden zusätzlich mit Opioiden versorgt (normale Kognition: 17 % mit Opioid; milde kognitive Beeinträchtigung: 75 %; Verdacht auf Demenz: 56 %). Ein Patient mit chronischen Schmerzen erhielt seine Vormedikation Hydromorphon 24 mg/Tag. Die anderen Patienten erhielten Tilidin (100 bis 300 mg/Tag).

### Schmerzangabe mittels numerischer Ratingskala (NRS)

Die mittlere postoperative Schmerzintensität (NRS; MW ± SD) aller Patienten betrug 4,0 ± 1,6. Die Patienten mit normaler Kognition zeigten eine mittlere postoperative Schmerzstärke von 4,8 ± 1,8 (NRS), die Patienten mit leichter kognitiver Beeinträchtigung von 3,3 ± 1,5 und Patienten mit Verdacht auf Demenz von 3,8 ± 1,3. Am ersten postoperativen Tag lag der NRS-Mittelwert ± SD aller Patienten bei 4,1 ± 1,8, am zweiten postoperativen Tag bei 3,5 ± 0,7 und am dritten postoperativen Tag bei 4,0 ± 1,4. Die berichteten Schmerzintensitäten unterschieden sich nicht zwischen den Kognitionsgruppen und den ersten drei postoperativen Behandlungstagen (ANOVA; *p* = n. s.).

### Schmerzempfindungsskala (SES)

Der Summenscore für die affektiven Deskriptoren betrug im Mittel ± SD 15,3 ± 10,9, für die sensorischen Deskriptoren 10,7 ± 4,9. Es konnten keine statistisch signifikanten Unterschiede für die sensorischen oder affektiven Summenscores der Schmerzempfindungsskala nachgewiesen werden (ANOVA; *p* = n. s.).

Für die Faktorenanalyse der SES-Summenscores für den sensorischen Teil der Schmerzempfindungsskala ergab sich mit einem dreifaktoriellen Modell die beste Varianzaufklärung (74,4 %). Hier brachte der Faktor „oberflächlicher Schmerz“ mit den dazugehörigen Begriffen „schneidend“, „stechend“, „heiß“ und „durchstoßend“ 29,7 % Varianzaufklärung, der Faktor „Tiefenschmerz“ mit den Begriffen „klopfend“, „brennend“ und „pochend“ ergab 24,6 % sowie der Faktor „intensiver Schmerz – oberflächlich und tief“ mit den Begriffen „reißend“ und „glühend“ insgesamt 20,1 % Varianzaufklärung. Bezüglich der Aufklärung an der Gesamtvarianz der Faktorenanalyse des affektiven Teiles der Schmerzempfindungsskala wurde mittels eines Zweifaktoren-Modells eine Prozentzahl von 81,5 erreicht. Faktor 1 lässt sich hier als „allgemeine affektive Attribute“ („quälend“, „grausam“, „unerträglich“, „mörderisch“, „elend“, „schauderhaft“, „scheußlich“, „marternd“, „furchtbar“, „heftig“) beschreiben und Faktor 2 als „affektives Attribut mit motorisch hemmender Assoziation“ („lähmend“). Für den Faktor 1 ergab sich eine Varianzaufklärung von 59,1 %, für den Faktor 2 von 22,4 %.

### Kognitive Fähigkeiten der Patienten

Der mittlere DemTect-Score (±SD) betrug 10,1 ± 4,5 Punkte. Von 19 Patienten lagen bei sechs (31,6 %) normale kognitive Fähigkeiten vor, bei vier Patienten (21,1 %) ergaben sich Hinweise auf leichte kognitive Einschränkungen, bei neun Patienten (47,4 %) ergab sich der Verdacht auf Vorliegen einer Demenz. Das Fehlen von Interaktionen in der ANOVA weist darauf hin, dass die Zugehörigkeit zu einer Kognitionsgruppe unabhängig vom Alter der Patienten war.

### Postoperatives Schmerzempfinden und QST

**Figure 1:**
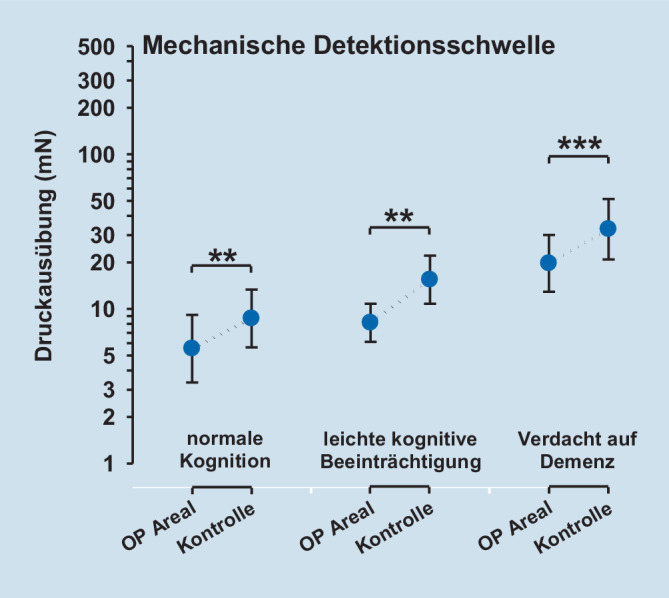

**Figure 2:**
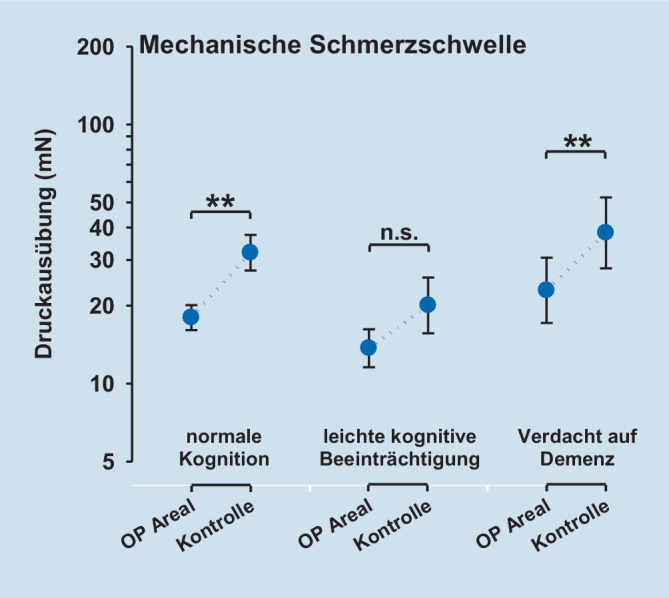

Die postoperative Schmerzempfindlichkeit – gemessen mittels QST – unterschied sich nicht zwischen den Kognitionsgruppen (Tab. 2, Abb. 1, 2, 3 und 4). Die QST-Parameter Druckschmerzschwelle (Tiefenschmerzempfindlichkeit; PPT), oberflächlicher mechanischer Schmerz nach Nadelreiz (MPT) und die oberflächliche Sensibilität für leichte Berührungsreize (MDT) zeigten jedoch hoch signifikante Unterschiede zwischen der operierten Seite und der gesunden Seite (ANOVA PPT: F = 47,9, *p* < 0,001; MPT: F = 20,5, *p* < 0,001; MDT: F = 49,6, *p* < 0,001). Für alle Parameter zeigte sich über der operierten Seite eine signifikant gesteigerte Empfindlichkeit. Für das Vibrationsempfinden (VDT) konnten keine Unterschiede zwischen operierter und gesunder Extremität nachgewiesen werden (ANOVA; *p* = n. s.). Die Ergebnisse sind in Tab. 2, sowie den Abb. 1, 2, 3 und 4 dargestellt.

**Table 2:** 

	Faktor 1: Vergleich operierte vs. Kontrollseite		Faktor 2: Kognitionsgruppe (normal, MCI, Demenz)		Interaktion Faktoren 1 * 2	
QST-Parameter	F‑Wert	*p*-Wert	F‑Wert	*p*-Wert	F‑Wert	*p*-Wert
PPT	47,9	< 0,001	0,23	n. s.	0,59	n. s.
MDT	49,6	< 0,001	1,08	n. s.	0,23	n. s.
MPT	20,5	< 0,001	2,41	n. s.	0,46	n. s.
VDT	5,0	< 0,05	0,44	n. s.	0,06	n. s.

Legende: *PPT* pressure pain threshold (Druckschmerzschwelle), *MDT* mechanical pain detection threshold (taktile Detektionsschwelle), *MPT* mechanical pain threshold (mechanische Schmerzschwelle), *VDT* vibration detection threshold (Vibrationsempfinden), *MCI* mild cognitive impairment (leichtes kognitives Defizit)

**Figure 3:**
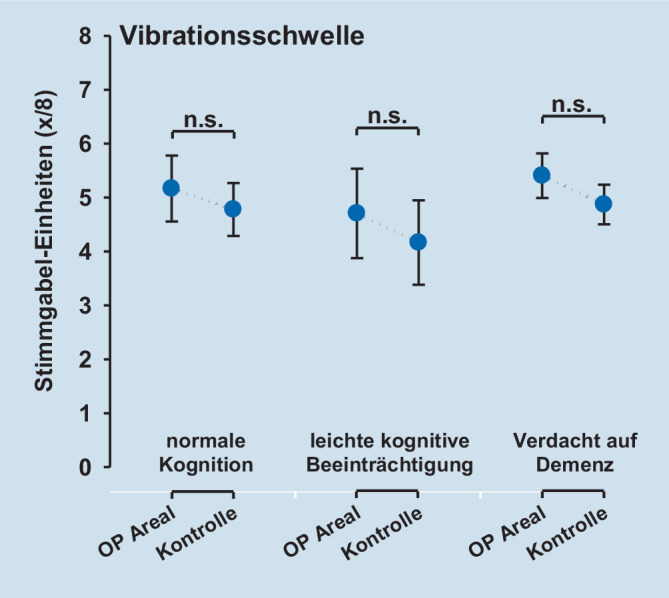

**Figure 4:**
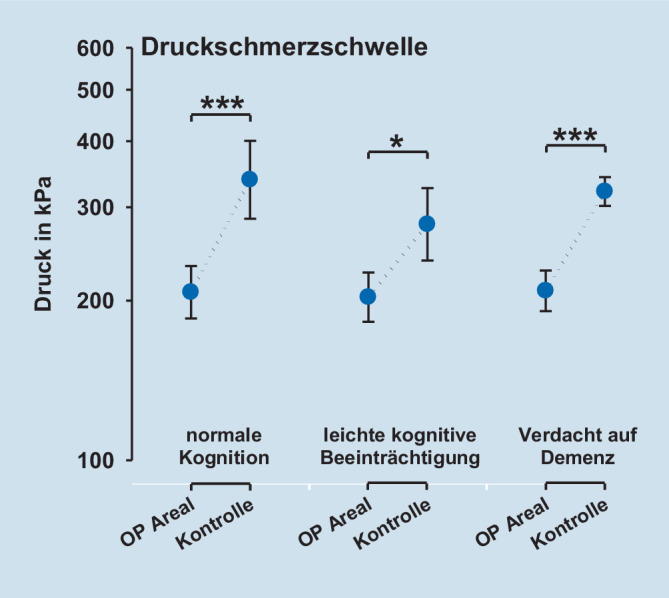

## Diskussion

Wesentliche Befunde der vorliegenden Studie weisen darauf hin, dass sich das postoperative Schmerzerleben zwischen Patienten mit normaler und deutlich eingeschränkter Kognition nicht unterscheidet. Speziell die Wahrnehmung evozierter Schmerzreize im Rahmen der quantitativen sensorischen Testung zeigte keine Unterschiede zwischen den Kognitionsgruppen. Unmittelbar nach Operation waren die affektiven Schmerzqualitäten stärker als die sensorischen ausgeprägt und nahmen im Verlauf des zweiten und dritten postoperativen Tags deutlich ab. Die quantitative sensorische Testung zeigte mechanische Hyperalgesien im operierten Areal, die mit dem Konzept einer zugrundeliegenden peripheren und am ehesten sekundären zentralen Sensibilisierung der Gewebe nach Operation vereinbar sind.

### Postoperative Durchführbarkeit kognitiver und sensorischer Tests nach Femurfraktur

Wie wichtig es ist, das Ausmaß postoperativer Schmerzen systematisch zu erfassen, wurde zuletzt in einer großen Kohortenstudie deutlich, in der die Intensität der Schmerzen mittels numerischer Ratingskala am ersten postoperativen Tag bei 179 verschiedenen Op.-Prozeduren erfasst wurde. Interessant war, dass gerade bei „kleineren“ Eingriffen wie zum Beispiel Appendektomie, Tonsillektomie und Hämorrhoidektomie ein unerwartet hoher postoperativer Schmerzlevel vorlag [16].

In der vorliegenden Studie war es möglich, unabhängig von Attributen wie Alter oder kognitiven Fähigkeiten, einfachere Befragungen wie die Erfassung einer numerischen Schmerzschätzung durchzuführen. Der DemTect als bereits im klinischen Alltag etabliertes Instrument zur Einschätzung der kognitiven Fähigkeiten eines Patienten konnte hier ohne Einschränkungen bei allen Patienten angewendet werden. Auch bei den differenzierteren Fragebögen wie der Schmerzempfindungsskala waren die Patienten mit leicht eingeschränkten kognitiven Fähigkeiten und mit Verdacht auf Demenz in der Lage, ihr sensorisches und affektives Schmerzerleben genau zu differenzieren. Diese Fähigkeit zur Schmerzbeurteilung spiegelte sich auch bei der Untersuchung mittels QST wider. Auch hier kam es weder zu alters- noch kognitionsbedingten Schwierigkeiten bei der Durchführung. Allen Patienten war es postoperativ möglich, die vorgesehenen Untersuchungen durchzuführen. Dies bestätigt frühere Untersuchungen, die nachweisen, dass von bis zu mittelgradig dementen Patienten Berührungsreize und Schmerzreize differenziert beurteilt werden können [3, 14]. Diese Befunde belegen die Indikation für eine vorausschauend geplante und regelhafte postoperative Schmerzerhebung (z. B. NRS) zur Sicherstellung einer angemessenen pflegerischen Betreuung und Schmerzbehandlung [24, 35].

### Postoperatives Schmerzerleben und medikamentöses Management

In der hier vorliegenden Studie zeigte sich kein signifikanter Zusammenhang zwischen postoperativ berichteter Schmerzstärke und kognitivem Status bei vergleichbarer analgetischer Therapie. Das postoperative Schmerzempfinden von Patienten mit kognitiver Beeinträchtigung unterschiedlichen Grades und kognitiv unbeeinträchtigten Patienten unterschied sich nicht signifikant. Auffällig war, dass anders als in der Literatur berichtet die Gruppen mit kognitivem Defizit häufiger Opioide in Ergänzung zu Nichtopioiden erhielten. Die kognitiven Einschränkungen waren also in unserem Kollektiv kein Prädiktor eines eingeschränkten Schmerzmanagements, was wir auf die etablierten Schmerzstandards im Studienzentrum zurückführen.

### Postoperatives affektives und sensorisches Schmerzerleben

Der Kognitionsstatus hatte keinen Einfluss auf das beschriebene affektive oder sensorische Schmerzerleben. Vor diesem Hintergrund erscheint die aus der Literatur bekannte Versorgungsrealität problematisch, die zeigt, dass demente Patienten weniger Schmerzmittel erhalten als solche mit normalen kognitiven Fähigkeiten [6, 30], auch im postoperativen Setting [42]. Eine Ursache könnte eine unzureichende und inadäquate Schmerzeinschätzung bei Patienten mit Demenz durch die betreuenden Ärzte und/oder das Pflegepersonal sein [33]. Zum anderen könnte eine unzureichende oder auch missverständliche Artikulation der Schmerzen durch die Patienten selbst eine Rolle spielen [24]. Schmerzäußerungen beispielsweise durch Vokalisationen werden häufig als postoperatives Delir oder Agitation fehlinterpretiert und könnten statt zu einer adäquaten Schmerztherapie zu sedierenden Maßnahmen wie der Gabe von Neuroleptika führen [19].

### „Sensory profiling“ mittels QST nach operativer Versorgung einer Femurfraktur

In der quantitativen sensorischen Testung (QST) zeigte sich, dass die operierte Seite im Vergleich zur Gegenseite eine Überempfindlichkeit für mechanische Reize aufwies. Nur über dem operierten Gebiet zeigten sich eine Druckhyperalgesie sowie eine Nadelreiz-Hyperalgesie. Dieser Befund unterschied sich von Ergebnissen der Gruppe von Kehlet, die eine erhöhte Tiefensensibilität, nicht aber eine Hyperalgesie für kutane Reize finden konnten [1]. Im Unterschied zu unserer Studie wurde für die kutane Reizung elektrischer Strom verwendet, der unter Durchdringung der Haut ebenfalls tiefere Gewebeschichten erreichte. Interessanterweise zeigte unsere Studie auch für nichtschmerzhafte Reize mit „von Frey“-Filamenten eine Hyperästhesie, die über A‑beta-Fasern vermittelt wird, während das ebenfalls peripher über A‑beta-Fasern vermittelte Vibrationsempfinden sich nicht zwischen der Op.-Seite und dem unbehandelten Kontrollareal unterschied. Dieser Befund weist darauf hin, dass leichte taktile Reize und Vibrationsreize trotz gleichem peripherem Fasersystem möglicherweise zentral über zwei verschiedene Projektionswege zum Thalamus weitergeleitet werden. Möglicherweise wird nur ein System im Rahmen des postoperativen Geschehens sensibilisiert, während das andere unbeeinflusst bleibt. Eine weitere Erklärung könnte sein, dass das Vibrationsempfinden mehr die Sensibilität tieferer Gewebe (protopathisch) reflektiert, während die oberflächliche Berührungsempfindlichkeit der Haut (epikritisch) besser durch den Parameter MDT repräsentiert wird. MDT mag damit stärker durch Umwelteinflüsse und Alterung der Haut als VDT beeinflusst werden. Zusätzlich mag es für die mechanische Detektionsschwelle mit von Frey-Filamenten eine Rolle spielen, dass die Erfassung dieses Parameters ein diffiziler Akt ist, der mehr als die Bestimmung der Vibrationsschwelle vom Kognitionsstatus abhängen könnte, was sich in einem diskreten Trend zu höheren Schwellen bei abnehmendem Kognitionsstatus andeutete. Auch sollte beachtet werden, dass VDT gegenüber dem DFNS-QST-Protokoll nicht über einer knöchernen Prominenz erfasst wurde.

### Vom klinischen Zeichen zum neurobiologischen Mechanismus

In der Literatur wurde bereits in Tierexperimenten gezeigt, dass nach bestimmten konditionierenden Reizen unterschiedliche neurobiologische Schmerzmechanismen induziert werden können. Im Rahmen von Entzündungsmodellen nach Injektionen von beispielsweise Capsaicin konnte eine gesteigerte Aktivität von zentralen „wide dynamic range“-Neuronen (WDR-Neuronen) im Hinterhorn des Rückenmarks nachgewiesen werden. Kardinalsymptom dieser zentralen Sensibilisierung ist eine mechanische Hyperalgesie für Nadelreize (Pinprick-Hyperalgesie) [43]. Hier können aber auch andere zentrale Mechanismen eine Rolle spielen [32].

Die Hyperalgesie für Nadelreize über der nicht durch die Operation betroffenen Haut muss als auf die Haut übertragene Hyperalgesie interpretiert werden („referred pain“-Konzept) [18]. Dieses Phänomen muss per Definition als zentral vermittelt angesehen werden – am ehesten im Sinn einer sekundären Hyperalgesie nach Sensibilisierung von WDR-Neuronen im Hinterhorn des Rückenmarks. Interessanterweise findet sich im Op.-Bereich auch eine taktile Hyperästhesie für leichte Berührungsreize („von Frey“-Filamente), die am ehesten ebenfalls zentral vermittelt ist. Dieses Phänomen einer sekundären mechanischen Hyperästhesie wurde bisher nicht beschrieben. In der Literatur wird in experimentellen Schmerzmodellen nach Capsaicin-Injektion das Phänomen einer sekundären taktilen Hypoästhesie bei gleichzeitiger Nadelreiz-Hyperalgesie beschrieben [28]. In diesem Modell liegt jedoch kein so ausgeprägter peripherer nozizeptiver Input vor wie nach der hier beschriebenen operativen Versorgung einer Femurfraktur. Neben den zentralen neuroplastischen Veränderungen im Schmerzsystem können auch periphere Sensibilisierungsprozesse für die lokal gesteigerte Schmerzempfindlichkeit verantwortlich sein. In dem oben beschriebenen Entzündungsmodell nach intrakutaner Capsaicin-Gabe kommt es primär zu einer peripheren Sensibilisierung von Nozizeptoren am Ort der Injektion, die zu einer lokalen Hitzehyperalgesie führen kann. Der TRPV1-Ionenkanal (Vanilloid-Rezeptor 1) hat eine wesentliche Bedeutung für die Entstehung der Hitzehyperalgesie, da er über eine spezifische Bindungsstelle (z. B. für Capsaicin) verfügt, zum anderen aber auch über eine erhöhte Temperatur aktiviert wird. Auch nach Schnittverletzungen kommt es zu einer lokalen Überempfindlichkeit gegenüber Hitzereizen (primäre Hitzehyperalgesie). Dies wurde nicht nur im Tiermodell bei Mäusen nachgewiesen [34], sondern auch im humanen Surrogatmodell [9]. Verantwortlich hierfür ist eine gesteigerte Erregbarkeit von Aδ- und C‑Faser-Nozizeptoren. Möglicherweise kann die Absenkung der Erregungsschwelle nozizeptiver C‑Fasern zu einer erleichterten Aktivierbarkeit führen und damit den Ruheschmerz triggern. Als weiteres Symptom einer peripheren Sensibilisierung wird in der Literatur eine Druckhyperalgesie beschrieben [21, 26]. Im getesteten Patientenkollektiv konnte dieser Befund einer gesteigerten Druckempfindlichkeit tieferer Gewebe reproduziert werden, was indirekt auf das wahrscheinliche Vorliegen einer primären, postoperativen peripheren Sensibilisierung hinweist. Diese und der damit verbundene gesteigerte sensorische Input führen vermutlich sekundär zu einer zentralen Sensibilisierung oder anderen zentral neuroplastischen Veränderungen, die ursächlich für die oben beschriebene Nadelreizhyperalgesie sein könnten.

### Limitierungen

Aus der vorliegenden Studie lassen sich keine Generalisierungen vornehmen, da im Rahmen dieser Pilotstudie nur eine kleine Patientenpopulation untersucht wurde (*n* = 19). Es kann letztlich nicht mit Sicherheit ausgeschlossen werden, dass die fehlenden Gruppenunterschiede durch diese kleine Fallzahl (zu geringe statistische Power) bedingt sind. Wünschenswert wäre eine Folgestudie mit größerer Fallzahl, um die hier gewonnenen Ergebnisse verifizieren zu können und mehr über das postoperative Schmerzerleben von Patienten mit eingeschränkten kognitiven Fähigkeiten zu erfahren. Dabei sollte auch beachtet werden, dass etwa die Dosierung von Schmerzmitteln wie Opioiden bestmöglich vergleichbar zwischen diesen Kognitionsgruppen ist. In unserer Studie wurden die Patienten mit normaler Kognition weniger häufig postoperativ mit Opioiden behandelt. Eine weitere Limitierung war die dem Eingriff geschuldete operationsnahe Erfassung der Vibrationsschwelle (VDT) über der Haut fernab von einer knöchernen Prominenz. Diese Abweichung vom Original-QST-Protokoll des DFNS führte zu VDT-Werten um 5/8, was sich jedoch nicht wesentlich von altersentsprechenden Referenzwerten etwa des Knöchels unterscheidet.

## Fazit und Ausblick

Es zeigte sich, dass die postoperative Schmerzstärke innerhalb der Patientengruppe (*n* = 19) unabhängig vom kognitiven Status war. Im sensorischen und affektiven Schmerzerleben ergeben sich im SES-Fragebogen (Schmerzempfindungsskala) zwischen den untersuchten Patientengruppen mit normaler Kognition bis hin zum Verdacht auf Demenz keine signifikanten Unterschiede. Die quantitative sensorische Testung (QST) weist nur über dem operierten Areal eine mechanische Überempfindlichkeit aus. Konkret zeigte sich dort neben einer Nadelreiz- und Druckhyperalgesie eine taktile Hyperästhesie für „von Frey“-Filamente. Das Vibrationsempfinden unterschied sich nicht zwischen operierter Seite und dem Kontrollareal. Die gefundene Druckhyperalgesie im operierten Gebiet ist vereinbar mit dem Konzept einer peripheren Sensibilisierung im nozizeptiven System. Diese periphere Sensibilisierung induziert möglicherweise sekundär auch eine zentrale Sensibilisierung. Passend zu dieser Annahme findet sich das klinische Zeichen einer Nadelreizhyperalgesie über der Haut des operierten Gebiets, die am ehesten zentral vermittelt ist – ebenso wie eine taktile Hyperästhesie, die nur zentralnervös erklärt werden kann und in dieser Form noch nicht beschrieben ist.

Insgesamt weisen alle Befunde dieser Feasibility-Studie darauf hin, dass Betroffene mit kognitiven Defiziten ein unverändertes Schmerzerleben und auch eine vergleichbare Reagibilität ihres nozizeptiven Systems aufweisen. Hieraus leitet sich der auch medizinethisch bedeutsame Anspruch ab, die gleichen hohen Ansprüche an eine optimale postoperative Schmerztherapie auch für Menschen mit Demenz zu stellen. Prinzipiell sind auch Studien mit anderen operativen Prozeduren für Menschen mit kognitivem Defizit wünschenswert.
